# Microscopic Dynamics Controls Coupling and Cluster Formation in Brush Particle Solids

**DOI:** 10.1021/acs.macromol.5c02236

**Published:** 2026-01-08

**Authors:** Qiqi Li, Jirameth Tarnsangpradit, Katarzyna Biniek-Antosiak, Yu Cang, Jiajun Yan, Jianan Zhang, Krzysztof Matyjaszewski, Bartlomiej Graczykowski, Michael R. Bockstaller, George Fytas

**Affiliations:** † Planck Institute for Polymer Research, Ackermannweg 10, Mainz 55128, Germany; ‡ Department of Materials Science and Engineering, 6612 Carnegie Mellon University, 5000 Forbes Avenue, Pittsburgh, Pennsylvania 15213, United States; § Faculty of Physics and Astronomy, 49562 Adam Mickiewicz University, Uniwersytetu Poznanskiego 2, Poznan 61-614, Poland; ∥ School of Aerospace Engineering and Applied Mechanics, Tongji University, Zhangwu Road 100, Shanghai 200092, China; ⊥ Department of Chemistry, Carnegie Mellon University, 4400 Forbes Avenue, Pittsburgh, Pennsylvania 15213, United States; # Institute of Electronic Structure and Laser, FORTH, N. Plastira 100, Heraklion 70013, Greece; ● Department of Molecular Physics, Faculty of Chemistry, Lodz University of Technology, Żeromskiego 116, 90-924 Łódź, Poland

## Abstract

Thermodynamics-based models predict the structure of polymer-grafted nanoparticles (PGNs) as well as their assembly behavior based on geometric parameters such as particle size, degree of polymerization, and density of grafted chains. The role of microscopic polymer dynamics, such as the mobility of repeat units in the melt state, in the evolution of the structure and properties remains unknown. Brillouin light spectroscopy (BLS), due to its capability to concurrently discern the local and global elastic properties of PGN assemblies, enables the probing of microscopic processes, such as brush interdigitation, sensitive to the annealing of the assembly. For poly­(methyl methacrylate) (PMMA)-grafted silica (SiO_2_) PGNs in the dry powder state and annealed above the glass transition temperature, BLS revealed fully reversible local elasticity, indicative of limited interdigitation between adjacent PGNs. This contrasts with polystyrene (PS)–SiO_2_ analogs that displayed ready (and irreversible) fusion of brush layers during annealing. The retardation of brush interdigitation in PMMA-grafted systems is surprising, given the similar thermomechanical properties of both polymers, and is rationalized as the consequence of higher friction between PMMA repeats compared to PS. Microscopic dynamics thus has a profound impact on the kinetic path of structure (and property) evolution and thus should be considered during the processing of PGNs into functional hybrid materials.

## Graphical abstract

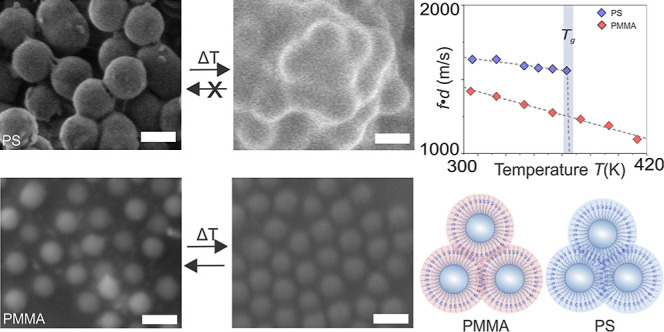

## 1 Introduction

Polymer-grafted nanoparticles (PGNs, aka “brush particles”) have emerged as a platform for the bottom-up fabrication of functional hybrid materials that derive unique property profiles such as increased dielectric barrier properties, thermal conductivity, subdiffusive thermal transport, selective gas transport, and impact resistance from the uniform microstructure and brush characteristics.^1–9^ More recently, unexpected bandgap behaviors were demonstrated in PGN assembly structures that provide new opportunities to control the flow of visible light and hypersound.^10–12^ Harnessing the unique property profiles of PGN-based materials requires an understanding of the respective roles of brush architecture and film microstructure on physical properties. Brush particle characteristics are commonly interpreted based on “geometric” parameters such as the degree of polymerization, *N*, dispersity *D̵*, grafting density, σ, and core size *R*_c_. Since the stretching energy per chain peaks at N* = (2√3 + 3)­ρ_K_ *R*_c_/σ (with ρ_K_ being the Kuhn monomer number density), PGN segments are considered “densely packed” (thus allowing for only limited interdigitation of grafted chains) for *N*_K_ < N*, with *N*_K_ denoting the number of Kuhn monomers per chain. Segments for which *N*_K_ > N* are considered to be in a “melt-like” state that supports interdigitation between adjacent brush chains.^13–15^ In the solution and melt state, visible light and X-ray scattering studies confirmed hard-sphere and soft (star-like) interactions for PGNs in the dry and semidilute brush regime, respectively.^5,8,13,15^ In films assembled from PGNs, a brush interdigitation zone of thickness *h*_int_ = *b*(*N*_K_ – *n*_dry_)^1/2^ was shown to promote a polymer-like elastic modulus and toughness (*b* is the segment length and *n*_dry_ is the number of segments in the dry brush regime).^5,14,16^ Material systems for elucidating structure–property relations of PGN solids have primarily been based on silica (SiO_2_) particles grafted with homo- and copolymers that are amenable to grafting-from modification via surface-initiated reversible deactivation radical polymerization.^17–20^ Silica is often chosen as a model system due to its versatile surface chemistry and stability of the Si–O–C coupling chemistry that prevents degradation of SiO_2_–based PGNs during thermal annealing. For the study of brush particle solids, atactic polystyrene (PS) and poly­(methyl methacrylate) (PMMA) have been most widely used because of their amorphous structure and comparably high glass transition temperature (*T*_g,PS_ ∼ 102 °C, *T*_g,PMMA_ ∼ 107 °C) that render the resulting materials model systems for establishing structure–property-processing relations.^21^

However, while atactic PS and PMMA share aspects such as disordered structure, similar glass fragility, and thermomechanical properties, both polymers differ in their dynamical characteristics. In particular, at otherwise identical conditions, the (zero-shear rate) viscosity of PMMA in the melt state exceeds the respective value of PS by more than 3 orders of magnitude.^22,23^ This has been attributed to a smaller packing length of PMMA (*p* = 0.36 nm) as compared to PS (*p* = 0.40 nm), which is the range over which the density near a monomer is dominated by monomers of the same chain and the associated reduction of the entanglement molecular weight (*M*_e_ ∼ *p* ^3^) for PMMA (13.6 kDa) compared to PS (18.1 kDa)^24^ as well as the significantly larger molecular friction between MMA repeats.^25^ The slower dynamics of PMMA could impact the equilibration kinetics of films, thus resulting in more pronounced trapping of nonequilibrium, low-entropy stretched conformations during film casting.^26,27^ Further, the increased friction between chains could prolong the time needed to facilitate brush interdigitation, thus resulting in more process-dependent properties of PGN solids.

This is illustrated in Figure 1 that depicts the change of microstructure with thermal annealing of SiO_2_–PMMA (σ = 0.5 nm^–2^, *N* = 1244) assemblies fabricated by precipitation of PGNs from poor solvent conditions (i.e., by rapid addition of excess amounts of methanol to 2% suspensions in toluene). Analysis of particle center-to-center distances revealed a PGN size of ∼213 nm in the pristine state (Figure 1a), close to the estimated pristine PNG size of 209 nm (Table 1). Thermal annealing for 24 h at *T* = 120 °C resulted in the reduction of the average PNG size to 191 nm (Figure 1b). This is close to the estimated reduction of the center-to-center distance upon filling of interstitial spaces (*d*_a_ ∼ 0.9 *d*_i_) and did not suggest a change in the brush layer structure other than the mentioned volume reduction of PGN assemblies upon thermal annealing. This contrasted with the irreversible “fusion” of brush layers that was observed previously in SiO_2_–PS systems.^28^ The contrasting behavior displayed by both brush systems suggests differences in structure formation that could impact the properties of materials assembled from both systems. Thus, understanding the role of kinetics and metastability on the structure of PGN assemblies is essential for the fabrication of PGN solids with deliberately controlled structure (and property) profiles.

**Figure 1:**
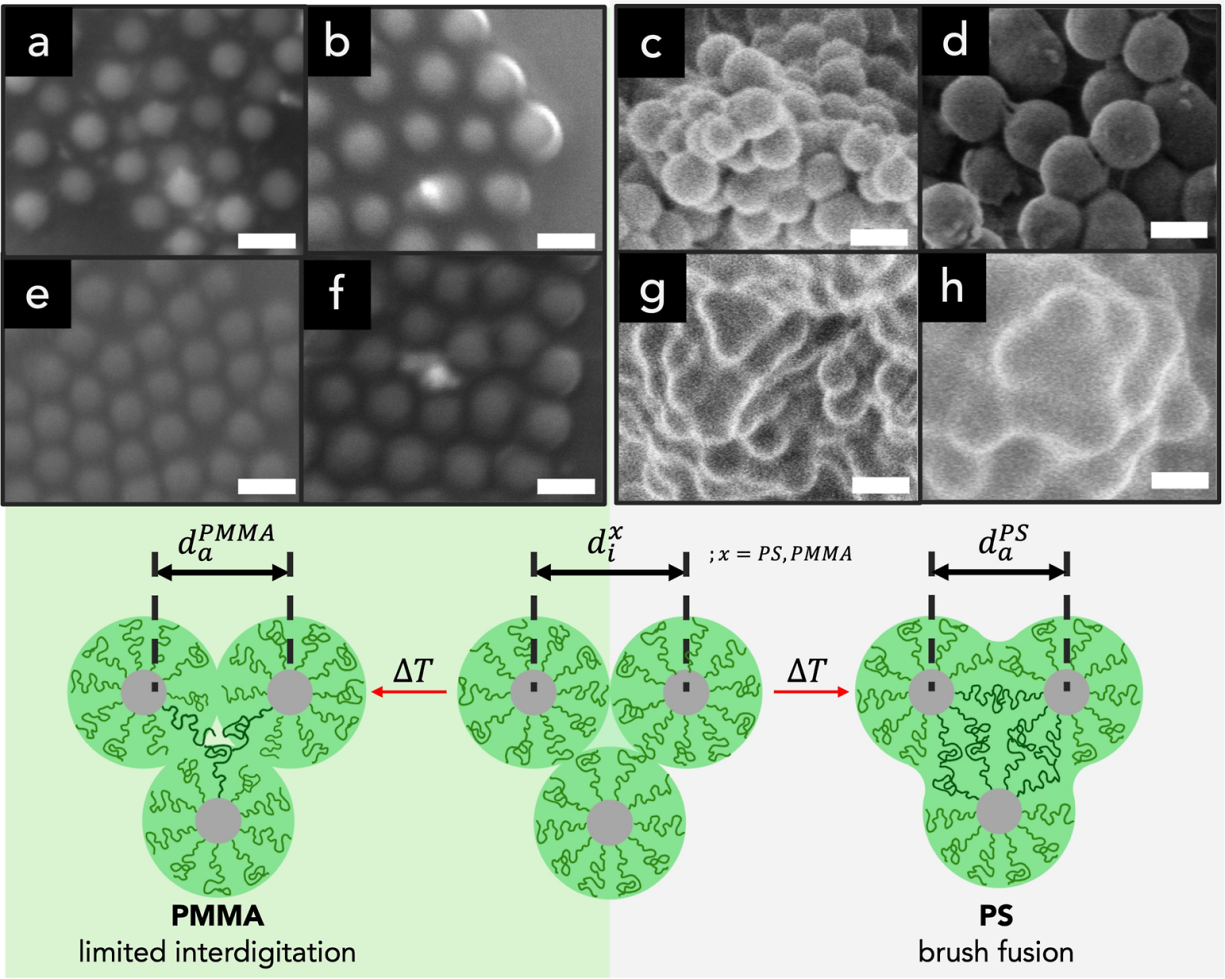
Scanning electron micrographs comparing structure transitions in SiO_2_–PMMA and SiO_2_–PS brush particle clusters before (a–d) and after (e–h) thermal annealing at *T* = 120 °C for 24 h. Images show SiO_2_–PMMA: *N* = 533 (a,e) and *N* = 1244 (b,f); SiO_2_–PS: *N* = 530 (c,g) and *N* = 1300 (d,f). Grafting density is σ ≈ 0.5 nm^–2^ for all systems. The scale bar is 200 nm. The average SiO_2_–PMMA size is determined as 191 nm (*N* = 533) and 213 nm (*N* = 1244). Images in panels c, d, g, and h represent unpublished data of SiO_2_–PS materials, which are reported in ref 28, for comparison. Images show more profound fusion in PS-brush systems as compared to PMMA analogs. The schemes illustrate the observed differences in structure evolution.

**Table 1:** Molecular Characteristics of the PGN Samples

sample-ID	ϕ_SiO_2__ from TGA	*N*	*D̵*	σ/nm^–2^	*d*/nm	*h*/nm	*h*_dry_ (*h*_int_/2)/nm
SiO_2_–PMMA-254	0.67	254	1.15	0.53	147 (149)	17 (14.7)	12.4 (2,3)
SiO_2_–PMMA-337	0.59	337	1.14	0.56	158 (159)	22 (19.4)	17.0 (2.4)
SiO_2_–PMMA-553	0.46	553	1.24	0.55	187 (176)	34 (27.8)	24.8 (3.0)
SiO_2_–PMMA-1244	0.30	1244	1.50	0.50	234 (209)	57 (44.7)	40 (4.7)

In this contribution, we examined the effect of graft composition of PGN solids at similar grafting density on the PGN interface region between individual brush particles via analysis of the vibration spectra of SiO_2_–PMMA PGN powder samples that were prepared by precipitation from aqueous solution following an analogous procedure as previously described for SiO_2_–PS.^29^ The vibration spectrum of nanoparticles is sensitive to the length scale and elasticity of core and shell components, as well as to the “state-of-coupling” that depends on the interdigitation between brush canopies of adjacent particles (which is expected to be limited for *N*_K_ < N*). This renders Brillouin light scattering (BLS) as a suitable technique to monitor brush interdigitation in PGN assembly structures. In the case of SiO_2_–PS PGN powder, the limited interdigitation in samples (prepared by precipitation from dilute solution) did allow the recording of individual brush particle vibration spectra.^28,30^ The latter became undetectable after heating the powder samples above the *T*_g_ of the grafted chains due to increasing interdigitation between adjacent brush particles. Interestingly, despite the similar molecular characteristics (*N*, *D̵*, σ) and *T*_g_ of the present SiO_2_–PMMA PGN systems compared to the previously investigated SiO_2_–PS, PMMA–PGNs featured a distinctively different temperature dependence of vibration spectra. We deliberately utilized dry powder PGN systems to avoid convoluting effects resulting from solvent volatility and preference. Powder samples were prepared by precipitation of brush particles after rapid addition of nonsolvent (methanol) to PGN dilute (<0.1%) suspensions in toluene and subsequent drying in vacuum. Vibration spectra of PGN powders were recorded by BLS^30–34^ at different temperatures below and above the *T*_g_ of PMMA. The elasticity of PGN clusters and individual PGN, which was deduced from the low-frequency dipolar and high-frequency quadrupolar vibrational modes, revealed PGN glass mobility and reversibility upon heating and cooling, in stark contrast to the results on SiO_2_–PS analogs,^28^ and consistent with a more limited graft interpenetration in the case of SiO_2_–PMMA.

## 2 Materials and Methods

### 2.1 Materials

Silica particles with ATRP initiating sites (SiO_2_–Br) were prepared as reported.^29^ Ethyl α-bromoisobutyrate (98%, Sigma Aldrich), anisole (99%, Aldrich), tetrahydrofuran (THF, 99%, VWR), methanol (99%, VWR), tris­(2-dimethylaminoethyl) amine (Me_6_TREN, 99%, Alfa), copper­(II) bromide (CuBr_2_, 99%, Aldrich), tin­(II) 2-ethylhexanoate (Sn­(EH)_2_, 95%, Aldrich), and *N*,*N*-dimethylformamide (certified, Fisher Chemical) were the materials used. Monomers: methyl methacrylate (MMA, 99%, Aldrich) was purified by passing through a column filled with basic alumina to remove the inhibitor. Alumina was used (basic, Super I, 50–200 μm, Sorbtech).

### 2.2 Synthesis of Brush Particles

The procedure followed the process used by Lee et al.^21^ Initiators (SiO_2_–Br), monomer MMA, solvents (anisole), CuBr_2_, and Me_6_TREN were mixed in the appropriate molar ratio (Schlenk flask) and subsequently degassed by bubbling with nitrogen. The conversion was monitored by ^1^H NMR. The final products were precipitated in cold methanol and, after dialysis, dissolved and stored in THF. A summary of the brush particle systems is presented in Table 1.

### 2.3 Size-Exclusion Chromatography

Grafted polymer characteristics including the number-average molecular weight (*M*_n_) and dispersity (*M*_w_/*M*_n_) were determined by using size-exclusion chromatography (SEC). To perform SEC, the grafted chains were cleaved from the silica core surface by etching with HF for 24 h. The etched samples were then neutralized with ammonium hydroxide and purified by passing through basic alumina and a 0.45 μm PTFE filter before SEC. Linear PMMA was used as the standard for calibration, and toluene was used as a standard for each measurement.

### 2.4 Thermogravimetric Analysis

The inorganic content φ_SiO_2__ and grafting density of the PGN were calculated based on the thermogravimetric analysis (TGA) results obtained using a TA Instruments TGA 550 under an air atmosphere. The heating steps were performed as follows: (1) equilibrate at room temperature; (2) jump to 120 °C; (3) hold at 120 °C for 10 min; (4) ramp up the temperature from 120 to 800 °C at 20 °C/min; (4) isothermal at 800 °C for 5 min. The data collected were analyzed by using TA Universal Analysis. *f*_SiO_2__ was determined from the remaining weight divided by the weight at the end of step (3) at 120 °C. σ_s_ was calculated from

$$\sigma_{s}=\frac{\left(1-f_{{SiO}_{2}}\right)N_{Av}\rho_{{SiO}_{2}}d}{6f_{{SiO}_{2}}M_{n}}$$

where *N*_Av_ is Avogadro’s number, ρ_SiO_2__ is the mass density of the silica particle (2.2 g·cm^–3^), *d* is the diameter of the SiO_2_ particles, and *M*_n_ is the number-average molecular weight of the grafted polymer brushes.

### 2.5 Differential Scanning Calorimetry

Thermal analysis was performed by using a TA Instruments DSC-Q20. Three heating–cooling cycles were performed from −50 to 160 °C with a ramp rate of 10 °C/min. The heat flow and derivative heat flow results were analyzed by using TA Universal Analysis to obtain the glass transition temperature of the PGN.

### 2.6 Sample Preparation

Film samples: all films were prepared via solvent casting at room temperature on a substrate of 2 × 2 cm^2^ borosilicate glass coverslip. The substrate was cleaned with isopropyl alcohol and was blown dry prior to the film preparation. Particle brushes in toluene solutions were used to prepare the films. The obtained thickness of the film was approximately 15 μm.

Powder samples: powder samples were prepared by precipitating PGN solutions (dissolved in toluene) in methanol. The precipitates were separated and annealed under vacuum at 120 °C for 24 h.

### 2.7 Scanning Electron Microscopy

SEM images of the PGN before and after annealing were obtained from a Tescan Mira 3 FEG SEM. SEM samples were prepared by precipitating diluted PGN solutions (dissolved in toluene) in methanol. The precipitates were then deposited onto a clean Silicon wafer substrate. Thermal annealing was performed for the annealed samples at 120 °C for 24 h. The particle diameter was measured using the software package ImageJ.

### 2.8 Transmission Electron Microscopy

TEM images collected using an FEI Tecnai F20 TEM/STEM instrument were used to determine the particle diameter (*d*) and brush height (*h*). TEM samples were prepared by drop-casting PGN solutions (∼10 mg/mL in toluene) onto the carbon-coated TEM copper grid. The samples underwent thermal annealing at 120 °C overnight to ensure full solvent removal.

### 2.9 Brillouin Light Spectroscopy

BLS is a powerful noncontact and nondestructive optical technique to probe the elastic vibrations of submicrometer particles at GHz frequencies.^30–34^ Utilizing the photoelastic interactions between incident light and thermally activated phonons, BLS records the spectrum of the inelastically scattered light by phonons with wave vector *q*. In the case of localized phonons (vibrations), their frequencies are *q*-independent, and the spectra are recorded at a back-scattering geometry utilizing a high-resolution tandem Fabry–Perot interferometer (JRS Instruments).

## 3 Results and Discussion

### 3.1 Brush Particle Characteristic Length Scales

The PGN material systems consisted of PMMA-grafted SiO_2_ with a core radius *R*_c_ ≈ 60 nm and varying *N* at roughly constant high grafting density (σ ≈ 0.55 nm^–2^) and dispersity (*D̵* ≈ 1.2). A summary of all PGN sample characteristics is given in Table 1. Based on the PGN diameter obtained from electron imaging (Figure S1), the computed value of the brush thickness, *h*, conformed to the scaling, *h* ∼ *N* ^0.76^, in good agreement with the calculated *h*_TLM_ ∼ *N* ^0.69^, which was based on the two-layer model (TLM) (Figure S2). To assess the capability of brush layers to interdigitate as schematically shown in Figure 1, we applied the TLM^14,15^ that assigns each brush layer a height *h* = *h*_dry_ + *h*_int_/2, where *h*_dry_ is the height of the dry (stretched) regime in which interdigitation is restricted and the interpenetration layer thickness, *h*_int_/2. Grafting density and space-filling conditions determine the crowding of chains and, thus, the structure and interactions of PGNs. In thermal equilibrium, the stretching free energy per chain, *E*_ext_ = (3/2)*k*_B_ *T*[*h* ^2^/(2*N*_K_ *b* ^2^)], depends on the thickness *h* = *R*_c_{[(1 + 3*ZN*_k_/(4π*R*_c_^3^ρ_Κ_)] – 1}^1/3^, where *Z* = 4π*R*_c_^2^σ is the number of chains grafted to each particle. The chain extension energy, *E*_ext_, depends on the ratio *y* ≡ 3*ZN*_k_/(4π*R*_c_^3^ρ_Κ_) of the polymer shell volume (*ZN*_k_/ρ_Κ_) to the core volume (4π*R*_c_^3^/3) and assumes a maximum value *E*_ext_^*^ ≈ 0.7*k*_B_ *T*σ*R*_c_/(*b* ^2^ρ_Κ_) at *y*_max_ ≃ 19.4, thus yielding N* and h* = √3 *R*_c_ ≃ 104 nm. The latter represents, essentially, a geometric crossover from a planar brush layer with *E*_ext_ ∝ *N* (*y* < 1) to a thick spherical polymer shell with *E*_ext_ ∝ *N* ^–1/3^ (*y* ≫ 1). An increase in grafting density and *R*_c_ enhances *E*_ext_, due to the increase of *h*, whereas the increase of ρ_Κ_ diminishes *E*_ext_, due to the reduction of the polymer shell volume.

The core–core distance *d* values are from TEM (Figure S1), whereas the values in parentheses denote the PGN diameter calculated from the two-layer model (TLM)^14^ using the values of monomer length, *l*_m_ = 0.16 nm, Kuhn length, *l*_K_ = 1.7 nm, *N*_K_ = monomers/Kuhn segment = 10.6, and ρ_K_ = 0.62 Kuhn segments/nm^3^.

Based on the values ⟨*R*_0_^2^⟩/*N* of the mean-square end-to-end distance per repeat of PMMA and PS chains,^24^ the monomer size of the two polymers was almost identical (∼0.16 nm), so the disparity in the packing lengths was due to different densities of the two polymers. Similarly, ρ_Κ,PMMA_ ≈ 0.62 nm^–3^ slightly exceeded ρ_Κ,PS_ ≈ 0.52 nm^–3^. It followed that for σ = 0.55 nm^–2^ (Table 1), N* ≈440 and *E*_ext_^*^ ≈ 13 k_B_ *T* for SiO_2_–PMMA, whereas for SiO_2_–PS, N* ≈ 370 and *E*_ext_^*^ ≈ 15 k_B_ *T*. For the SiO_2_–PMMA PGNs (Table 1), the value of *N*_K_ = *N*/*n*_K_, with *n*_K_ ≃ 10.6 being the number of monomers per Kuhn segment, ranged from 24 to 117, well below N*. Accordingly, the structures of all four PGNs should be dominated by extended polymer graft conformations. For comparison, the *N*_K_ of the SiO_2_–PS PGNs, previously examined,^10,11,28^ ranged from 11 to 122 (*n*_K_ ≃ 11.3), suggesting a similar extended state. For the four SiO_2_–PMMA PGNs, the interpenetrated layer ranged between 10%–15% of the total thickness and *E*_ext_ was between 1.7 kT and 3.2 kT for the *N* of Table 1. For SiO_2_–PS with *N* = 1300 and *R*_c_ = 57 nm, *h*_dry_ ≃ 52 nm, *h*_int_/2 ≃ 2.8 nm, and *E*_ext_ = 6.4 kT. We note that our description is only approximate since *E*_ext_ is only one part of the total free energy, which also includes other entropic and enthalpic contributions. However, within this approximation, we expected similar brush-like structures for both SiO_2_–PMMA and SiO_2_–PS PGN solids and thus a similar thermomechanical behavior, also revealed by molecular dynamics simulations,^35^ in contrast to that displayed in Figure 1. We attribute the reason to the sluggish kinetics of the SiO_2_–PMMA system, rather than thermodynamics, since in SiO_2_–PS PGN solids, interdigitation was observed.^28,35^ That kinetics can prevent interdigitation in brush systems is a major finding of our work.

### 3.2 Particle Vibration Spectroscopy

For homogeneous spherical colloids with dimensions less than the probing wavelength (∼π/*q*) of BLS, the lowest fundamental (Lamb) quadrupolar eigenmode probed by BLS occurred at *f*(1,2) = 0.85*c*_t,p_/*d*, where *n* = 1 and *l* = 2 denote the radial and angular momentum, respectively, and *c*_t,p_ is the transverse sound velocity within the particle.^31,32,34^ This interpretation was also shown to apply to core/shell colloidal architectures.^35^ Here, we utilized the same analysis for composite spherical nanoparticles to estimate the particle’s effective transverse sound velocity. From the measured *f*(1,2) eigenmode (24.3 GHz, Figure 2a), the transverse sound velocity of the SiO_2_ core was computed as *c*_t_ = (3490 ± 40) m/s using *d*_TEM_ = (122.6 ± 0.2) nm obtained from electron imaging. In contrast to the SiO_2_–PS PGN materials, the spectrum of the largest SiO_2_–PMMA-1244 displayed in Figure 2b is richer in features, as will be discussed below. The frequency, *f*(1,2) = 8.7 GHz, assigned to (1,2) mode according to theoretical calculations of the vibration spectrum of PGNs,^28^ yields the effective transverse velocity of this composite NP, *c*_t,p_ = 2400 m/s, assuming *d* = 234 nm from TEM (Table 1). This significantly exceeds the value estimated on the basis of the TLM model (*d* = 209 nm, Table 1). For the two high *N* PGNs, we used the spacing *d* from TEM consistently.

**Figure 2:**
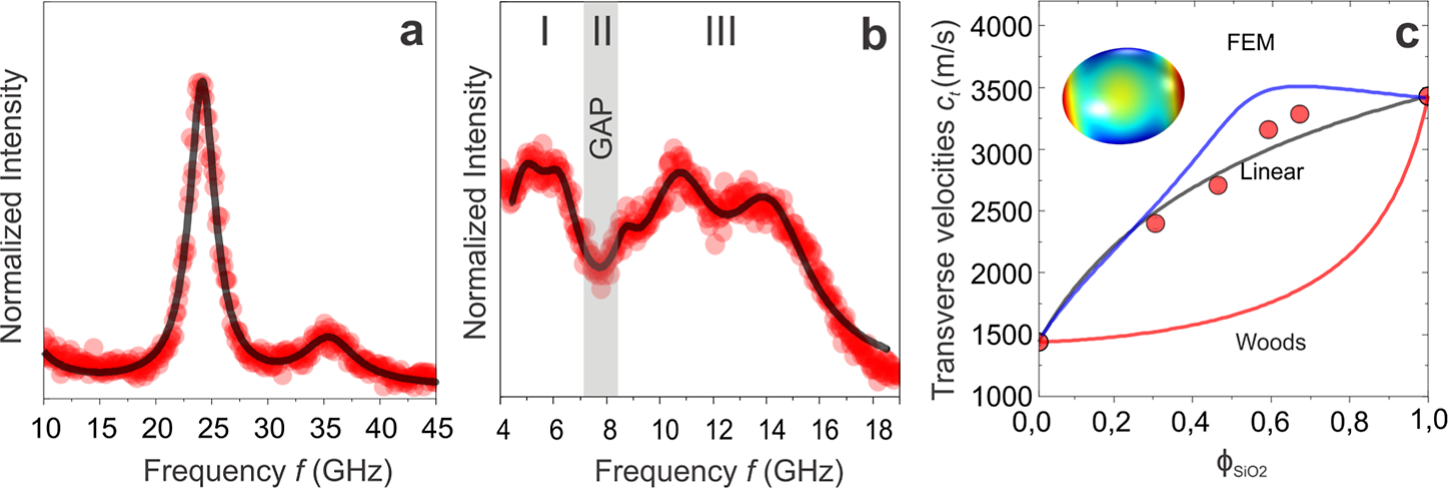
Vibration spectra of (a) bare SiO_2_ particles and (b) SiO_2_–PMMA-1244 PGN represented (solid lines) by Lorentzian line shapes. The vertical stripe denotes the frequency region of the propagation stopband falling between the frequency range (I) of interactions induced translational modes (1,1) in PGN clusters and the frequency range (III) localized vibration modes (1,2), (1,3) etc. (c) The effective transverse sound velocity *c*_t,p_ (symbols) in four SiO_2_–PMMA PGNs as a function of φ_SiO_2__ (Table 1) compared to the upper (black) and lower (red) bounds of the rule of mixtures and the FEM simulations (blue line). The inset shows the displacement field of (1,2) spheroidal eigen vibration calculated at φ_SiO_2__ = 0.6. The error in *c*_t,p_ amounts to ± 5%.

The variation of *c*_t,p_ with the core volume fraction φ_Sio_2__ (Table 1) shown in Figure 2c is much closer to the prediction of the upper (black) than the lower (red line) bound corresponding to the rule of mixtures for loading under constant strain and constant stress, respectively.^36^ This trend was expected for a spherical brush geometry, for which a part of the grafted chains is always oriented in the loading (displacement) direction. Finite element method (FEM) simulations (blue solid line in Figure 2c) further supported this assertion. The FEM model considered an individual core–shell particle composed of a spherical silica core covered by a PMMA shell of variable thickness. Both materials are homogeneous and elastically isotropic, with perfect mechanical coupling at the interface. We used the respective material characteristics as input parameters such as φ_SiO_2__, longitudinal and transverse velocities, and mass density, listed in Table S1. The model is based on classical elastodynamics and searches for undamped mechanical eigenmodes and vibrations and their eigenfrequencies. To evaluate the effective *c*_t,p_ of the composite particle, we utilized the solution corresponding to the (1,2) spheroidal Lamb mode (inset to Figure 2c) by recalling the formula known for the homogeneous NPs.^31,32,34^ Note that the model simplifies the shell structure of PGNs, which is inhomogeneous and does not involve particle interactions. Thus, the FEM predictions (blue line) are much closer to the linear rule of mixtures (black line) than Wood’s lower bound (red line in Figure 2c) over the entire composition range, in agreement with the BLS experimental *c*_t,p_ being closer to the upper bound limit.

In the case of the pristine SiO_2_ particles, there was no additional mode at frequencies lower than the quadrupolar *f*(1,2), while the higher-frequency eigenmode was assigned to the (1,3) mode.^31,32,34^ However, in the case of SiO_2_–PMMA PGNs, there was a low-frequency branch below *f*(1,2), which related to acoustic excitations of clusters of PGNs connected via, for example, van der Waals interactions.^33,34^ This mode was more discernible in the power spectra *I*(*f*) *f* ^2^ (Figure 3a,b), which corresponds to as-measured BLS spectra normalized by the phonon thermal population factor (1/*f* ^2^ at ambient temperature). The frequency of the dipolar, NP–NP interaction-induced, mode, *f*_int_(1,1) = (2*E*_eff_/ρ_eff_)^1/2^(π*d*)^−1^ was determined by the effective elastic constant, *E*_eff_ = ρ_eff_ *c*_[110]_^2^, with ρ_eff_ and *c*_[110]_ = πd*f*(1.1) $/\sqrt{2}$ being the density and the longitudinal sound velocity in a cluster of face-centered cubic (fcc) lattice; the prefactor (π $/\sqrt{2}$ ) depends on the cluster structure.^34^ We note that the assumption of a periodic (fcc) structure of PGN clusters simplified numerical calculations while providing close approximations to resonances in high-grafting density PGN assemblies that feature close-packing characteristics.^10^ The temperature dependence of *f*(1,2) and *f*_int_(1,1) should yield information on the variation of *c*_t_,_PMMA_ of the grafted PMMA (*c*_t_,_SiO_2__ is temperature-independent) and *E*_eff_ of the cluster, respectively.^33,34^ The former should reveal whether the thermal expansion of the PGN glass toward *T*_g_ of PMMA resembles that of the bulk PMMA, whereas the latter should relate to the strength of the PGN granular structure and the extent of graft interpenetration in the glassy and melt state. Finally, the unexpected increase of the intensity of the (1,1) branch with temperature (see Figure S3) suggests enhanced interactions of neighboring PGNs. As the spectrum is independent of the largest scattering wave vector at back-scattering (*q*_bs_ ≈ 0.035 nm^–1^) employed, the size of the interaction “cluster” is indeed of the order of core–core separation, implying interactions mediated via the PMMA grafts. In this context, we should also mention that an additional length scale of the BLS, the phonon mean-free path (≈1–2 μm), averages the local structures probed with a resolution π/*q*_bs_. Hence, the rich pattern of the (1,1) branch in SiO_2_–PMMA (Figures 2b and 4a) compared to SiO_2_–PS PGN^28^ brush implies different degrees of layer interdigitation in the two systems and provides additional insight into the origin of the different microstructures observed in PS-/PMMA-grafted systems (Figure 1).

**Figure 3:**
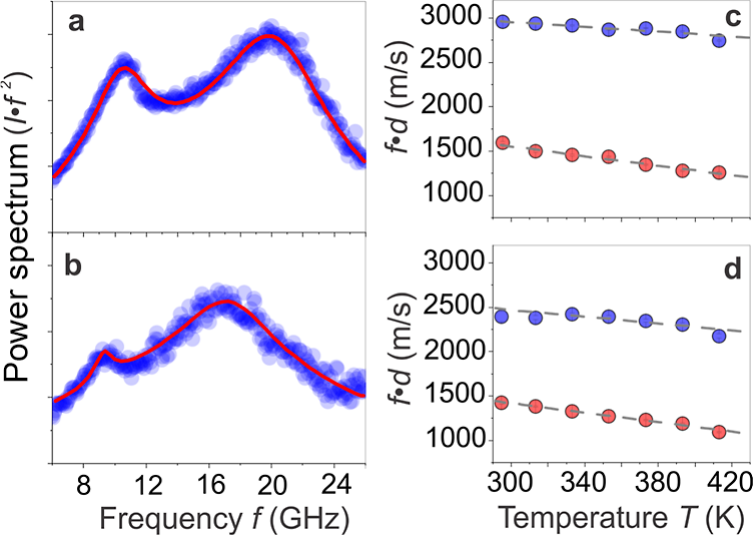
BLS spectra of SiO_2_–PMMA-254 (a) and SiO_2_–PMMA-337 (b) at 295 K recorded with a laser wavelength of 532 nm. The temperature dependence of the effective sound velocities (*fd*) for the interaction (*f*_int_ ∼ 9–10 GHz) (red symbols) and particle vibration (*f*_vib_ ∼ 17–19 GHz) (blue symbols) modes of SiO_2_–PMMA-254 (c) and SiO_2_–PMMA-337 (d) powder, using *d* = 147 nm and *d* = 158 nm, respectively. The presence of vibration modes above *T*_g_ indicates limited graft interpenetration insufficient to cause film formation. No *T*_g_ is discernible in *f*_int_(*T*).

**Figure 4:**
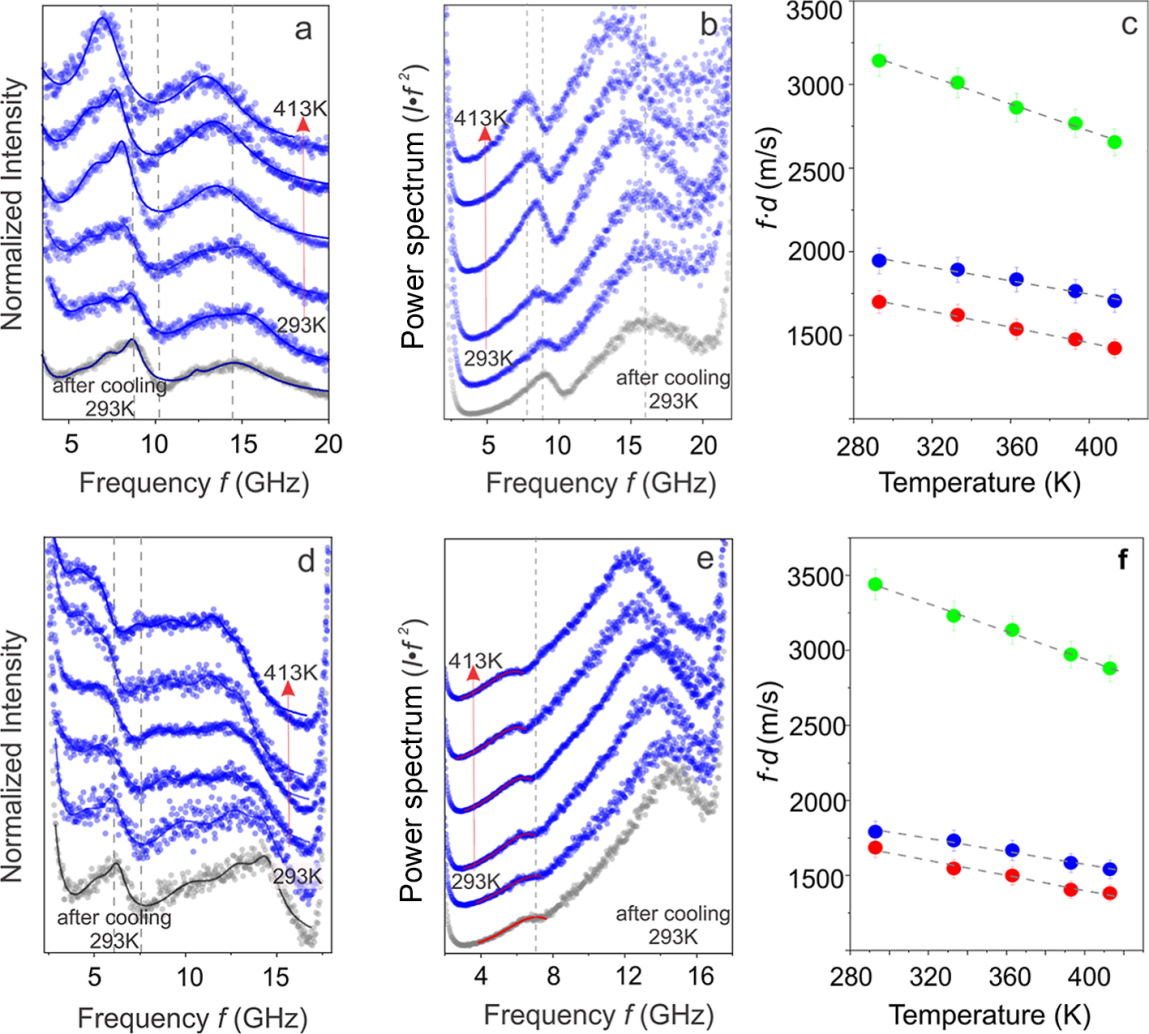
Vibration spectrum of SiO_2_–PMMA-553 (a) and the variation of the power spectra *I*(*f*)­x*f* ^2^ with temperature in (b) obtained from the intensity *I*(*f*) spectra of Figure S5 recorded at 532 nm. For SiO_2_–PMMA-1244, the intensity (d) and power (e) spectra of SiO_2_–PMMA-1244 at different temperatures. The temperature dependence of the three characteristic reduced frequencies (*f*_int_ *d* (red), *f*_dip_ *d* (blue), and *f*_vib_ *d* (green symbols) for SiO_2_–PMMA-553 (c) and SiO_2_–PMMA-1244 (f) obtained from the power spectra of (b,e). The dashed lines (in m/s) in (c): *f*_vib_ *d* = 4083­(1–9.3 × 10^–4^ T) (green); *f*_int_ *d* = 2250 (1–9.8 × 10^–4^ T) (red); *f*_dip_ *d* = 2400 (1–7.9 × 10^–4^ T) (blue) and In (f): *f*_vib_ *d* = 4273 (1–9.6 × 10^–4^ T) (green); *f*_int_ *d* = 2143 (1–1.0 × 10^–3^ T) (red); *f*_dip_ *d* = 2179 (1–8.9 × 10^–4^ T) (blue). The error in (c,f) *c*_t,p_ amounts to ± 3%.

### 3.3 Dispersion Characteristics of SiO_2_–PMMA in the Glassy and Melt State

For clarity, the dispersion characteristics of SiO_2_–PMMA PGN films in the glassy and melt state will be discussed separately for low-*N* (*N* = 254, 337) and high-*N* (*N* = 553, 1244) systems, respectively. The TLM diameters *d* for the two low-*N* PGN were close to the TEM spacing (Table 1). The power spectra of SiO_2_–PMMA-254 and SiO_2_–PMMA-337 powder at ambient temperature are shown in Figure 3a,b, respectively. For resolution of (1,1), we utilized the temperature-dependent power spectra, which led to a blue shift of (1,2) compared to the BLS intensity spectra (Figure S3). The variation of the particle transverse sound velocity, *c*_t,p_(*T*) ≈ *f*(1,2)*d*, and the cluster longitudinal sound velocity, *c*_c_(*T*) ≈ *f*(1.1)*d*, with temperature is depicted in Figure 3c,d for SiO_2_–PMMA-254 and SiO_2_–PMMA-337, respectively. Several findings are pertinent: (i) the response to thermal treatment is reversible as indicated by the reproducible results upon cooling (Figure S3), which suggests the absence of a contiguous film of PMMA grafts (Figure 1e,f). Note that for SiO_2_–PS PGN^28^ with similar molecular characteristics and PGN structure, polymeric (PS, PMMA) colloids,^33,34^ and SiO_2_/PMMA core–shell NPs,^37^ heating above the *T*_g_ of the polymer resulted in the irreversible “smearing” of the vibration spectrum due to the formation of a contiguous polymer film as a consequence of sufficient mixing in the contact surface between PGNs (Figure 1g,h). We recall that the fraction of intermixing in SiO_2_–PMMA PGN should be low due to the short interpenetration layer and the reduced mobility of PMMA chains in the melt state. (ii) Both the effective transverse *c*_t,p_ and the longitudinal *c*_c_ speed of sound decreased linearly with temperature, but the latter decreased at a higher rate. Specifically, for SiO_2_–PMMA-254, *f*(1,2)*d* = 3360­(1 – 4 × 10^–4^ T) m/s and *f*(1,1)*d* = 2365­(1–1.1 × 10^–3^) m/s; for SiO_2_–PMMA-337, the corresponding slopes, 6 × 10^–4^ K^–1^ and 1.5 × 10^–3^ K^–1^, assume higher values than for SiO_2_–PMMA-254 with lower *N*. (iii) Inspection of the shape of the (1,1) mode with increasing temperature revealed an increase of shape asymmetry for *N* = 254 compared to *N* = 337 (Figure S3), which was reversible for both systems. A rationalization of these findings follows next.

First, the reversible SiO_2_–PMMA PGN structure after heating above *T*_g_ that persisted in the melt state even at *T*_g_ + 40 K suggested either a deep trapped PMMA configuration or limited mixing between grafts of adjacent PGNs in the interpenetration regime, in contrast to SiO_2_–PS.^28^ We note that only a SiO_2_–PS PGNs with very low *N* = 130 and vanishingly small *h*_Int_/2 resembled the present case with a robust vibration spectrum.^28^ This supported the conclusion that the graft interdigitation was limited. Hence, the deep-trapping of PMMA grafts can be rationalized by the higher local friction of PMMA compared to PS. Second, the weaker *T*-dependence of the effective *c*_t,p_ suggested that the brush chains in SiO_2_–PMMA-254 form a lower-entropy glass (*E*_ext_ ≈ 1.7 k_B_ *T*) as compared to SiO_2_–PMMA-337 (*E*_ext_ ≈ 2.3 k_B_ *T*) analog, which displayed the anticipated decrease of *c*_t,p_(*T*) (Figure 3c), similar to bulk PMMA glass.^38^ Thus, increasing the *N* of grafted chains (i.e., SiO_2_–PMMA-337) resulted in more pronounced glass mobility along with stronger *T*-dependence of *c*_C_(*T*). This suggests that the entropy of the respective PGN assembly increases with *N* of the tethered chains and the associated increase of graft interdigitation (see the similar trend at even larger *N* in Figure 4c,f). It might also relate to a crossover from a planar brush layer to a thick spherical polymer shell for *N* ≥ 337. The stronger *T*-dependence of *c*_C_(*T*) than *c*_t,p_(*T*) is indicative of the different nature (cluster elasticity vs particle elasticity) of the two physical quantities. Remarkable is the reversibility of the “cluster” interactions upon cooling from *T* > *T*_g_. We note that this mode featured some dependence on the probing spot location, which was consistent with the expected heterogeneity of cluster structures across the sample area (Figure S4). This further supported the cluster nature of the mode.

The vibration spectrum of SiO_2_–PMMA-553 at 295 K in Figure 4a was, like the corresponding spectrum of SiO_2_–PMMA-1244 in Figure 2b, more feature-rich than for lower *N* analogs (Figure 3a,b). The fine structure revealed by the (1,1) branch for SiO_2_–PMMA with *N* = 533 and *N* = 1244 resembles the vibrational density of states (DOS). Assuming an fcc structure of the PGN clusters, three translational modes, i.e., two transverse and one longitudinal acoustic phonon, are expected for each PGN. In the case of an ill-defined scattering wave vector, as in the case of multiple light scattering, the three BLS quasi-peaks correspond to three van Hove singularities (region I in Figure 2b).^33^ The frequency of the (1,1) mode in the power spectrum (Figure 4b,e) is a measure of the interparticle interactions and enabled the estimation of the long-wavelength sound velocity in clusters. The detection of the fine structure, i.e., transverse mode singularities, requires both strong NP contacts and a translational order exceeding the phonon mean free path. For thermally populated GHz phonons at ambient temperature, the latter is of the order of few wavelengths. At backscattering, 2π/*q*_bs_ ≈ 180 nm, where *q*_bs_ is the largest magnitude of the wave vector. The fine structure of the (1,1) mode pointed to well-ordered PGN “clusters”. The increase in *N* and hence of *q*_bs_ *d* enabled the resolution of localized vibration modes with *l* > 2 (region III in Figure 2b).^32^ The regions of the propagating (I) and localized (III) modes are separated by the drop of the intensity *I*(*f*) at a frequency *f*_dip_ (*f*_dip_ ∼ 10.5 GHz in Figure 3a and *f*_dip_ ∼ 7.5 GHz in Figure 2b). This spectral feature is related to the region of phononic band gap,^39^ which occurred at *f*_dip_ ∼ *c*_eff_/*d*, where *c*_eff_ is the effective medium longitudinal sound velocity. Hence, *f*_dip_ should scale with *d* ^–1^ as seems to be the case for *N* = 553 (*d* = 187 nm) and *N* = 1244 (*d* = 234), for which the ratio of *f*_dip_ is about 1.4. Overall, the complexity of the low-frequency branch (region I in Figure 2b) suggests a nonperiodic short-range order, which is consistent with electron images such as those shown in Figure 1.

To investigate the glass mobility of the two systems with high *N*, we analyzed the temperature dependence of the power spectra in Figure 4b,e obtained from the corresponding *I*(*f*) spectra (Figures S5 and 4d), respectively, for SiO_2_–PMMA-553 and SiO_2_–PMMA-1244 below and above the *T*_g_ of bulk PMMA. The fine structure of the (1,1) branch smeared out with increasing temperature, and the evolution of the spectral shape was also manifested in the power spectra. The low-frequency peak was more pronounced for SiO_2_–PMMA-553 due to the deep intensity minimum compared to SiO_2_–PMMA-1244 with lower φ_SiO_2__ and therefore a narrower band gap width.^11^ From the temperature dependence of the power spectra (Figure 4b,e), the estimated reduced frequencies, *f*_int_ *d* (red), *f*_dip_ *d* (blue), *f*_vib_ *d* (green symbols), from the peak of (1,1), the minimum of DOS, and vibration branch are plotted as a function of temperature in Figure 4c,f. Note that the pattern of the PGN vibration modes was lost in the power spectrum presentation (the noise is magnified with increasing frequency) yielding a broad peak at ∼17 GHz (b) and 14.7 GHz (e) and *f*_vib_ *d* exceeded the transverse sound velocity *c*_t,p_ in Figure 2c; the temperature dependence of *f*_vib_ *d*, however, should represent that of *c*_t,p_(*T*). Following the trend in the low *N* systems (Figure 3c,d), *c*_t,p_ (*T*) for the two high *N* samples (Figure 4c,f) displayed a stronger temperature dependence (slope of 9.5 × 10^–4^ K^–1^) through the *T*_g_ of PMMA.

Both SiO_2_–PMMA and SiO_2_–PS PGNs possess similar brush structures with a low fraction of unperturbed chain segments in the interpenetration layer, implying limited interdigitation. Both systems feature similar *T*_g_ values and should thus display similar behavior in the solid and melt states. Yet, in spite of the expected limitation of chain interpenetration (short *h*_int_/2), interdigitation of grafted chains was readily observed^28,35^ for SiO_2_–PS (*N* = 1300), as indicated from the formation of a contiguous film above *T*_g_. Hardly identifiable individual SiO_2_–PS PGNs (Figure 1g,h) were also confirmed from the vanishing of eigen vibrations in the PGN vibration spectrum upon heating above *T*_g_.^28^ This suggested that polymer graft intermixing in the interpenetration region of SiO_2_–PS creates a cohesive network of PGNs. Unexpectedly, this is not the case for SiO_2_–PMMA PGNs (Figures 3 and 4), for which only a limited graft interdigitation is deduced from the vibration spectrum that is independent of annealing above *T*_g_ for all four graft lengths. Thus, in the case of PMMA, we concluded that kinetically arrested structures are formed that persisted across the relevant time scales even up to *T*_g_ + 40 K. An alternative interpretation, i.e., a reduction of interdigitation due to more bulky structure of MMA, was excluded based on the formation of contiguous films for (PS, PMMA) colloids^33,34^ and SiO_2_/PMMA core–shell NPs^37^ above the *T*_g_ of the polymer featuring craze formation.^17^ Hence, the BLS results presented above provide insight into the unexpected role of the chemical identity of the graft polymer composition on structure evolution in brush particle assemblies.

First, the frequency for all three elastic excitations, interaction and vibration eigenmodes, and the frequency at the intensity dip (corresponding to minimum DOS) in the two high *N* PGNs displayed a monotonic decrease with temperature through the *T*_g_ of PMMA (Figure 4). The rate of decrease for *f*_vib_ *d* (∼0.9 × 10^–3^ K^–1^) increased further than *c*_t,p_(*T*) for SiO_2_–PMMA-337 and even more than for SiO_2_–PMMA-254, which might support the computed higher entropy (5.4 k_B_ *T*, Section 3.1) glass for the high *N* PGNs. While *h*_int_/2 increases in absolute terms with *N* (Table 1), its contribution to the total thickness decreases, and therefore, there was no *T*_g_ discernible in the curves of Figure 4c,f, as in the case of the lower entropy glasses in Figure 3c,d. This could rationalize the robustness of the vibration spectrum to temperature variations also above *T*_g_, in contrast to the undetectable vibration spectrum of SiO_2_–PS analogs. We interpret the distinct behaviors of SiO_2_–PMMA PGNs due to the differences in local friction and entanglement molecular weight between PMMA and PS. The monomer friction at *T*_g_ is much larger (10^(5.54–2.06)^ ≈ 3000) for PMMA (Table 12-II in ref 25) as compared to PS. Moreover, the two polymers display different non-Arrhenius temperature dependence of the monomer friction coefficient (Figure S6). In the equilibrium melt state, the entanglement molecular weight has a profound impact on the dynamics of grafted chains.^40^ Notably, PMMA also exhibits sluggish surface dynamics compared with PS, which further facilitates the interdigitation in PS-grafted nanoparticles.^41,42^

Second, the low-frequency mode of the PGN clusters with different *N* displays a similar temperature dependence of the longitudinal sound velocity *c*_*c*_ ≈ d*f*_int_ (*T*) (Figures 2c,d and 3c,f) that indicates the softening of the cluster as a whole. An indication for the latter was the variation of the spectral pattern of the (1,1) branch with temperature, which is distinct in all four PGNs (Figures S3, 4, and S5). However, the reversibility of the cluster structure upon cooling from *T* > *T*_g_ is remarkable. Third, the *T*-dependence of *c*_L_(*T*) ∝ d*f*_dip_(*T*) reflects the variation of the longitudinal sound velocity within the cluster, which should be like d*f*_int_(*T*). The reversibility of d*f*_dip_(*T*) is consistent with the reversibility of the other two branches. The unexpected absence of a *T*_g_ in the temperature dependence of the SiO_2_–PMMA PGN vibrations (expressed in all three characteristic sound velocities, Figures 3 and 4) suggested their decoupling from the PMMA local segmental dynamics. The latter featured a cooperative rearrangement region (∼3 nm), which was manifested in the specific heat relaxation of the DSC, which revealed a *T*_g_ of ∼ 100 °C at a rate of 10 °C/min (Figure S7). Therefore, in contrast to the PS analogs, the temperature dependence of sound velocities in Figure 3c,d as well as Figure 4c,f should be determined by cohesive (entropic) forces rather than by a change in free volume at *T*_g_. In a similar context, glassy polymer brushes displayed a reduced thermal expansion as compared to films of similar thickness that renders the evidence of *T*_g_ for the former weaker.^43^ Considering the PMMA and PS PGN systems, the variation of the free volume with temperature should be even weaker in the kinetically trapped SiO_2_–PMMA PGNs. This suggested that kinetic parameters, previously not considered in the analysis of PGN assembly structures, present an additional dimension to rationalize and ultimately control processing structure–property relations in PGN assembly structures.^44,45^

## 4 Conclusions

Structure–property relationships in amorphous polymer-grafted nanoparticle (PGN) materials are often interpreted based on the two-layer model (TLM) that ascribes brush canopies a “dense/stretched” or “semidilute/relaxed” conformational state depending on geometric packing and free energy considerations. Accordingly, the TLM has been used to rationalize brush interdigitation, entanglement formation, and the related properties of self-assembled PGN solids. However, the applicability of the TLM depends on whether the kinetic parameters that govern structure formation enable the formation of close-to-equilibrium states that are described by thermodynamic models such as the TLM. In this contribution, BLS was used to reveal distinctive differences in the evolution of structure and interactions in poly­(methyl methacrylate) (PMMA)-grafted PGN assembly structures as compared to previously reported polystyrene (PS)-grafted PGN analogs. The sensitivity of BLS to both the local and bulk elastic properties of PGN assemblies reveals retardation of brush interdigitation and interstitial filling in PMMA–PGN materials, which alters PGN coupling and the associated low-frequency dispersion characteristics in PGN cluster structures. The observed difference is surprising, given the similar static and dynamic properties of PMMA and PS systems with a similar brush structure. We interpret the retardation of brush interdigitation and interstitial filling as the considerably sluggish dynamics of PMMA due to the increased friction between MMA repeats. The retardation of interstitial filling renders PMMA–PGN systems susceptible to the formation of frozen-in metastable states. Signatures of metastability are the absence of a *T*_g_ and the temperature dependence of the elasticity of PGN clusters in the BLS spectra. We note that DSC did in fact resolve *T*_g_, due to its sensitivity to the dynamic arrest of the PMMA segmental dynamics. The results therefore highlight the sensitivity of structure and properties of PGN-based materials to the dynamics on the repeat-unit level that will need to be considered during the processing of materials or when a comparison between materials with different compositions is to be made.

## Supplementary Material



## Notes

TEM images of materials, brush height analysis, intensity, power BLS spectra, and DSC analysis (PDF)

## ¶

School of Physical Science and Technology, ShanghaiTech University, Shanghai, 201210 China

## ∇

School of Chemistry and Chemical Engineering, Anhui University, Hefei 230601, China
