# Supplementary material for: Microscopic Dynamics Controls Coupling and Cluster Formation in Brush Particle Solids
Source: Macromolecules. 2026 Jan 8;59(2):653–62. doi: 10.1021/acs.macromol.5c02236 (PMC12854760; doi:10.1021/acs.macromol.5c02236)
Supplement: Supplementary file 1 [file ma5c02236_si_001.pdf]

Microscopic Dynamics Controls Coupling and Cluster Formation in Brush Particle Solids

Qiqi Li[1], Jirameth Tarnsangpradit[2], Katarzyna Biniek-Antosiak[3], Yu Cang[4], Jiajun Yan[5,¶], Jianan Zhang[2,#], Krzysztof Matyjaszewski[5], Bartlomiej Graczykowski[3], Michael R. Bockstaller*[2], George Fytas*[1,3,6]

[1]Planck Institute for Polymer Research, Ackermannweg 10, 55128 Mainz, Germany
[2]Department of Materials Science and Engineering, Carnegie Mellon University, 5000 Forbes Ave., Pittsburgh, Pennsylvania 15213, United States
[3]Faculty of Physics and Astronomy, Adam Mickiewicz University, Uniwersytetu Poznanskiego 2, 61-614 Poznan, Poland.
[4]School of Aerospace Engineering and Applied Mechanics, Tongji University, Zhangwu Road 100, Shanghai 200092, China
[5]Department of Chemistry, Carnegie Mellon University, 4400 Forbes Avenue, Pittsburgh, Pennsylvania 15213, United States
[6]Institute of Electronic Structure and Laser, FORTH, N. Plastira 100, Heraklion, 70013 Greece
¶) current address : School of Physical Science and Technology, ShanghaiTech University, Shanghai, 201210 China
#) current address : School of Chemistry and Chemical Engineering, Anhui University, Hefei 230601, China

Supporting Information

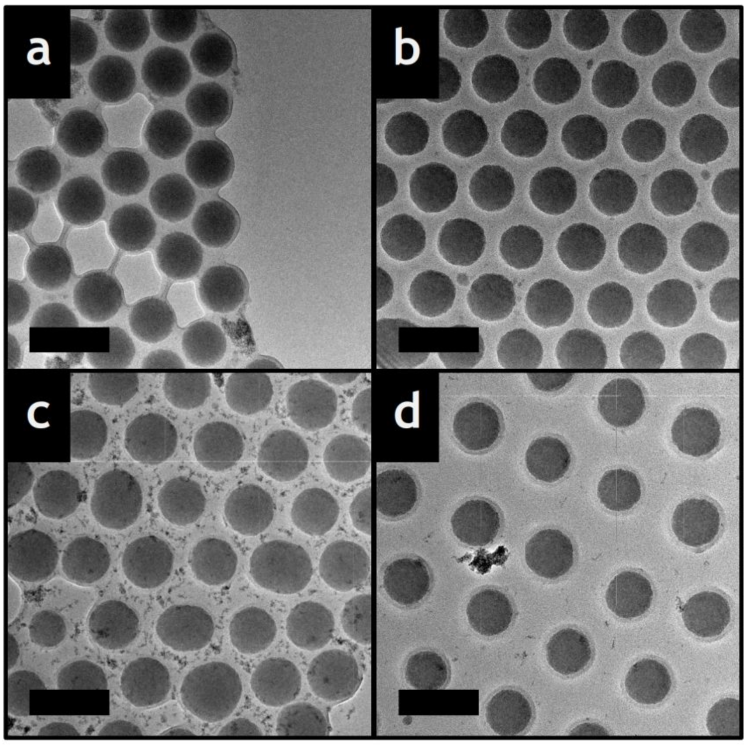

Figure S1. TEM images of SiO_2-PMMA-254 (a), SiO_2-PMMA-337 (b. SiO_2-PMMA-533(c) and SiO_2-PMMA-1244 (d). The scale bars are 200 nm.

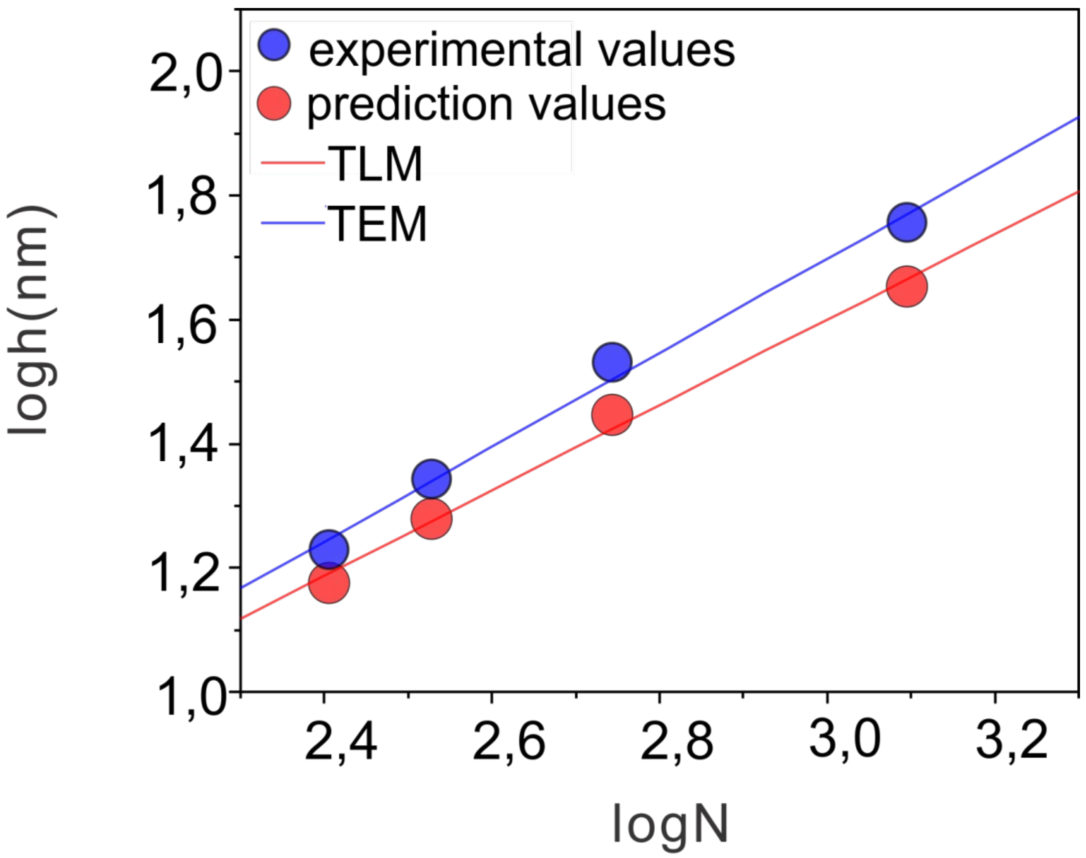

Figure S2. Log-log plot of the grafted polymer thickness, *h*, vs the degree of the polymerization of the PMMA grafts.$h \sim N^{0.76}$ for the experimental h obtained from TEM and $h \sim N^{0.69}$ the predicted values from TLM. Both scaling exponents indicate extended graft conformation. The thickness estimated from TEM amounts to about ±5%

Table S1. Sound velocities and density of the silica core and bulk PMMA

Material	c_t (m/s) (±1.5%)	c_l(m/s) (±1.5%)	ρ (g/cm^3)
SiO_2	3400	5390	2.0
PMMA	1440	2900	1.15

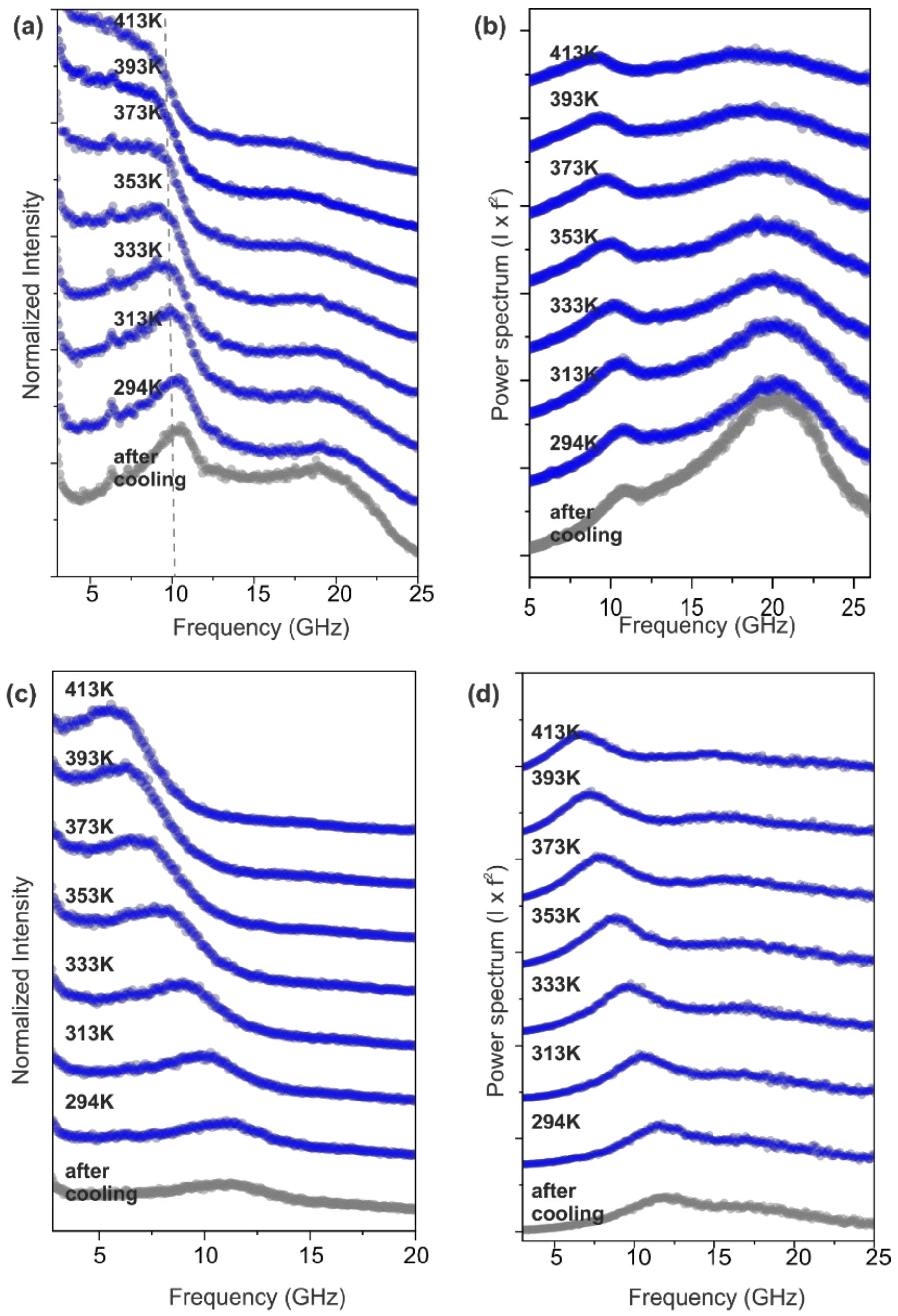

Figure S3. Intensity and power spectra of SiO_2-PMMA-254 (upper panel) and SiO_2-PMMA-337(lower panel) recorded at 532nm at different temperatures.

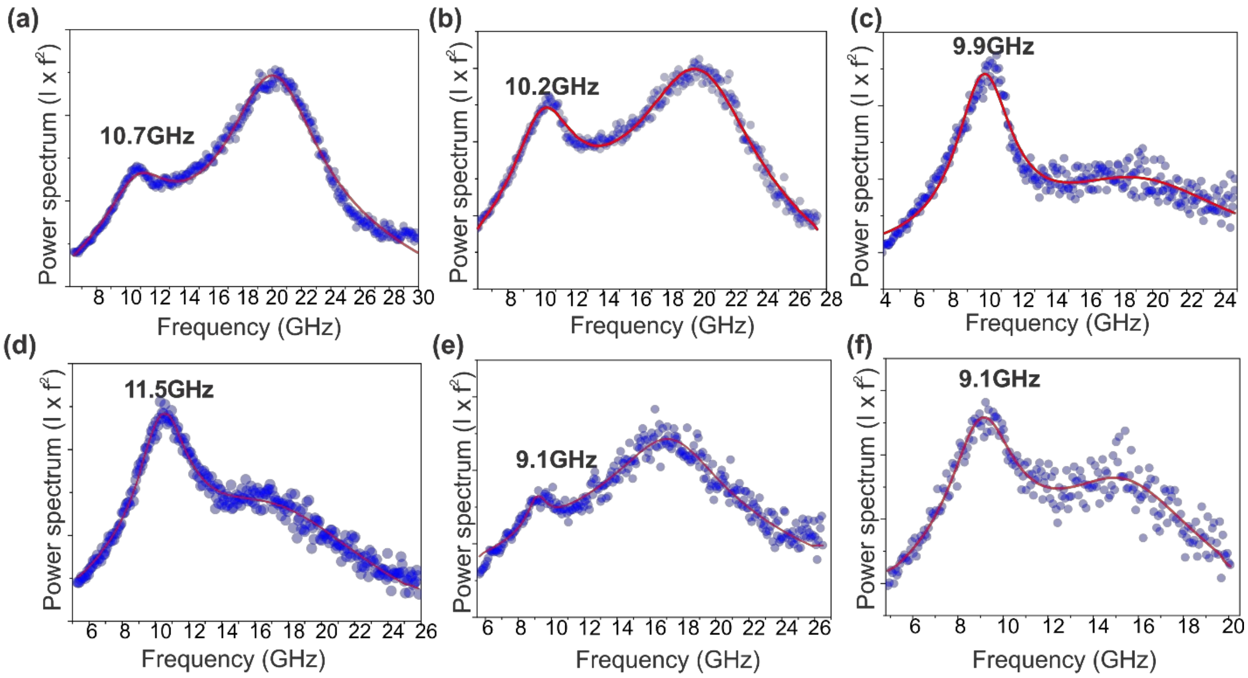

Figure S4. Upper Panel: Vibration spectrum of SiO_2-PMMA-254 at 295K recorded with laser wavelength 532nm and polarization VV (left) and VH (middle). Right panel: VV polarization and laser wavelength at 660nm. The slight variation of the $f(1,1)$ might relate to the cluster density. **Lower panel**: Vibration spectrum of SiO_2-PMMA-337 at 295K recorded with laser wavelength 532nm and polarization VV (left) and VH (middle). Right panel: VV polarization and laser wavelength at 660nm. The slight variation of the $f(1,1)$ might relate to the cluster density. The amplitude of quadrupolar eigenmode (1,2) depends on the magnification factor $q_{bs}d$, where $q_{bs}(=4\pi n/\lambda)$ and n is the refractive index and λ the laser wavelength [28]. Hence, this peak intensity is stronger at 532nm than at 640nm.

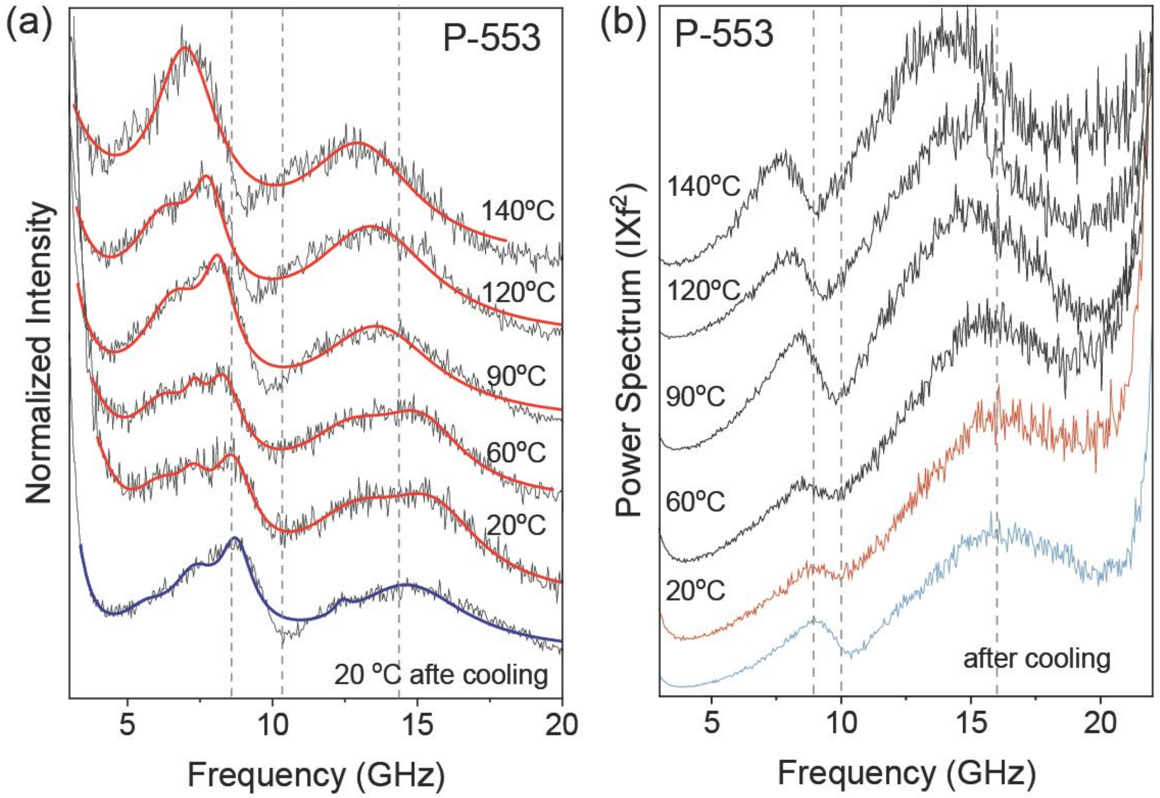

Figure S5. The intensity (a) and power (b) spectra of SiO_2-PMMA-553 (P-553) at different temperatures. Note the smearing of the fine structure in the intensity spectra with increasing temperature.

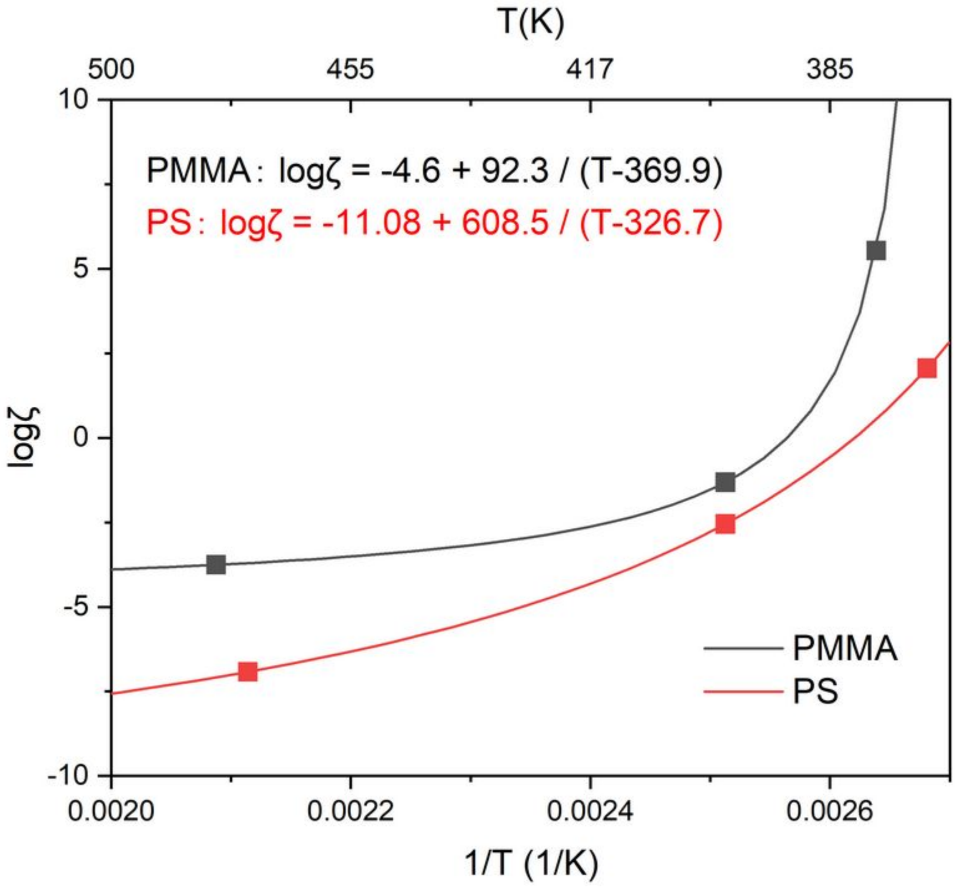

Figure S6. Temperature dependence of the monomer friction coefficient for bulk PMMA and PS (Table 12-II in ref.25). The solid lines denote the representation by a non-Arrhenius temperature dependence.

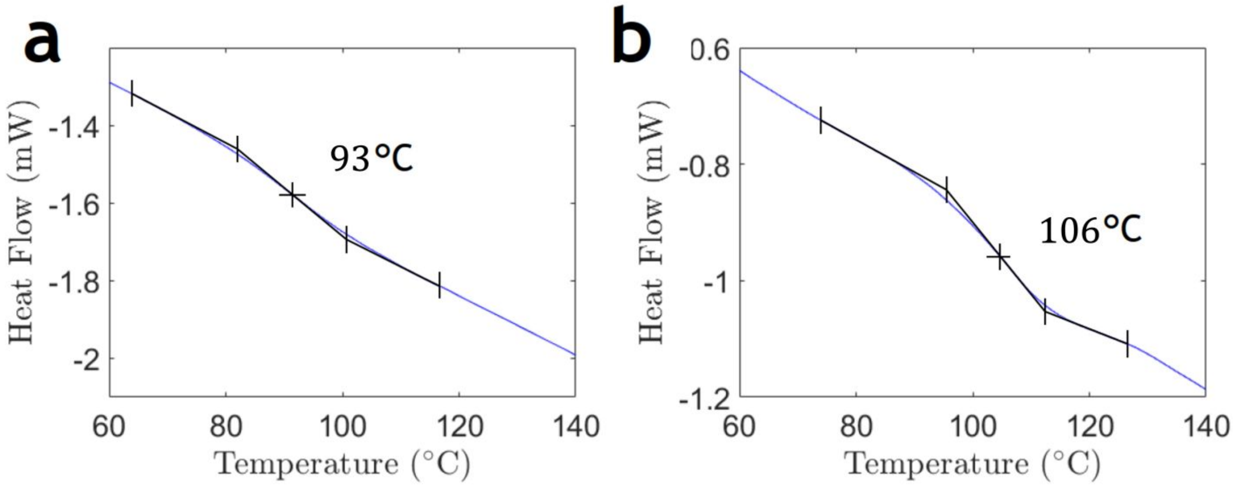

Figure S7. DSC traces for of SiO_2-PMMA-553 (a) and SiO_2-PMMA-1244 (b) taken at a ramp rate is 10 ^{o}C/min. The estimated glass transition temperature is listed in the plot.
